# Shared decision making and experiences of patients with long-term conditions: has anything changed?

**DOI:** 10.1186/s12913-018-3575-y

**Published:** 2018-10-10

**Authors:** Reem Kayyali, Shereen Nabhani Gebara, Iman Hesso, Gill Funnell, Minal Naik, Thuy Mason, Mohammed Ahsan Uddin, Noor Al-Yaseri, Umar Khayyam, Teebah Al-Haddad, Roshan Siva, John Chang

**Affiliations:** 1 School of Life Sciences, Pharmacy and Chemistry, Kingston University London, Penrhyn Road, Kingston upon Thames, KT1 2EE UK; 2 Croydon Health Services NHS Trust, Croydon University Hospital , 530 London Road, Croydon, CR7 7YE UK; 3 St. George’s Hospital, Blackshaw Road, Greater London, SW17 0QT UK

**Keywords:** Chronic obstructive pulmonary disease/COPD, Shared decision making/SDM, Healthcare professionals/HCPs, Patient centered care, Hospital discharge, Medicine optimization, Medicine use review/MUR

## Abstract

**Background:**

Medication problems among patients with long-term conditions (LTCs) are well documented. Measures to support LTC management include: medicine optimisation services by community pharmacists such as the Medicine Use Review (MUR) service in England, implementation of shared decision making (SDM), and the availability of rapid access clinics in primary care. This study aimed to investigate the experience of patients with LTCs about SDM including medication counselling and their awareness of community pharmacy medication review services.

**Methods:**

A mixed research method with a purposive sampling strategy to recruit patients was used. The quantitative phase involved two surveys, each requiring a sample size of 319. The first was related to SDM experience and the second to medication counselling at discharge. Patients were recruited from medical wards at St. George’s and Croydon University Hospitals.The qualitative phase involved semi-structured interviews with 18 respiratory patients attending a community rapid access clinic. Interviews were audio-recorded and transcribed verbatim. Thematic analysis using inductive/deductive approaches was employed. Survey results were analysed using descriptive statistics.

**Results:**

The response rate for surveys 1 and 2 survey was 79% (*n* = 357/450) and 68.5% (240/350) respectively. Survey 1 showed that although 70% of patients had changes made to their medications, only 40% were consulted about them and two-thirds (62.2%) wanted to be involved in SDM. In survey 2, 37.5% of patients thought that medication counselling could be improved. Most patients (88.8%) were interested in receiving the MUR service; however 83% were not aware of it. The majority (57.9%) were interested in receiving their discharge medications from community pharmacies. The interviews generated three themes; lack of patient-centered care and SDM, minimal medication counselling provided and lack of awareness about the MUR service.

**Conclusion:**

Although patients wanted to take part in SDM, yet SDM and medication counselling are not optimally provided. Patients were interested in the MUR service; however there was lack of awareness and referral for this service. The results propose community pharmacy as a new care pathway for medication supply and counselling post discharge. This promotes a change of health policy whereby community-based services are used to enhance the performance of acute hospitals.

**Electronic supplementary material:**

The online version of this article (10.1186/s12913-018-3575-y) contains supplementary material, which is available to authorized users.

## Background

Prescription of medications is the most common intervention in healthcare [1, 2]. However, medication problems occurring during the transition of care/after hospital discharge are well documented in the literature [3–5] and may lead to patient harm and hospital readmissions [5]. These difficulties are exacerbated because providers do not provide sufficient medication information to patients. The National Outpatient Survey (2011), found that only 43% of patients were confident that healthcare professionals (HCPs) were counselling them about all side-effects of their medications, (a decrease from 45% in 2009) [6].

Problems in medication usage are also documented in primary care especially among patients with long-term conditions (LTCs) [7]. These have been reported to negatively impact patients’ adherence and safety and lead to increased use of medical resources, such as physician visits, emergency department visits, hospital admissions and treatment failure [7]. An example of this would be chronic obstructive pulmonary disease (COPD), which is a chronic inflammatory condition that has been well documented in the literature to pose a high economic burden due to costly hospital admissions and medication wastage [8, 9]. Furthermore, poor adherence to medications and poor inhalation technique are widely reported problems among respiratory patients that lead to sub-optimal management and care, and increase the economic burden of COPD [9]. Therefore, some trusts such as Croydon University Hospital in the UK has established a rapid access clinic, referred to as “the respiratory HoT clinic” to improve the outcomes for COPD patients and prevent unnecessary hospital admissions. The service is managed by Croydon hospital respiratory team and provides the chance for patients to be seen by a specialist from secondary care in the primary care setting on the same day or next day, thus reducing the risk of accident and emergency visits due to sudden deterioration in their COPD condition [10].

Other measures to support LTCs management include medicine optimisation services offered by community pharmacists across many countries such as UK, United States, Canada and Australia [11, 12]. In England, cognitive services such as Medicine Use Review (MUR) and New Medicine Service (NMS) were introduced in the community pharmacy sector in 2005 and 2011, respectively as part of the government’s strategy to promote medicine optimisation and reduce medication wastage for patients suffering from LTCs [13]. According to the National Institute for Health and Clinical Excellence (NICE) guidelines [14], medicine optimisation requires a patient centred approach for safe and effective medicines use, in order to ensure that patients are getting the best possible outcomes from their medicines.

Shared decision making (SDM) is another approach that has been advocated by the UK government over the years [14] with the aim of encouraging patient involvement in care decisions during consultations between patients and HCPs to improve patients’ outcomes and quality of care [15], and promote medicine optimisation [14]. SDM is a fundamental component of patient-centred care [16]. The latter has been demonstrated to be beneficial in terms of improving patients’ outcomes, satisfaction, adherence to medications and disease burden [16–22].

This study aimed: first, to investigate hospital patients’ receipt and involvement in medication counselling, patients’ HCP preference for receiving medication information at the transition of care, and awareness of community pharmacy services such as the MUR. Second, to explore perceptions and experiences of patients with LTCs about the quality of care received in primary care and their awareness of the MUR service provided in the community pharmacy sector, focusing on COPD patients as an example.

## Methods

This is a mixed method research study involving quantitative cross-sectional surveys and qualitative semi-structured interviews with patients across South West London.

### The quantitative phase: Survey with patients

This phase was undertaken to address the first aim of this study and involved two cross-sectional surveys at two large South West London teaching hospitals: St George’s Hospital (6 wards) and Croydon University Hospital (2 wards). The two surveys were conducted sequentially in each hospital. For each survey, data was collected over 4 weeks at a rate of two days per week for each ward. Data collection started at St George’s Hospital, 6 medical wards were targeted and data collection took over 4 weeks for each ward. Once survey one was completed, data collection started for survey 2 using the same strategy. Thus data collection took 6 months for each survey, a total of one year for both surveys from December 2013 till December 2014. For survey one, screening of drug charts was also performed for each patient who completed the survey. This was conducted by the researcher pharmacists to check whether any new medication was added or any changes in drug dose, strength or formulation was made in relation to the main LTC they suffer from.

At the start of February 2015, data collection commenced at Croydon University Hospital for 2 medical wards and took over 4 months using the same strategy and was completed end of June 2015.

The first survey (see Additional file 1) was conducted among 357 patients at both hospitals and covered two areas: knowledge/experience/opinion of SDM and the patient’s involvement in decisions made about changes to their medication. The second survey was conducted among 240 patients at both hospitals and measured medication counselling received whilst in hospital and patient interest in receiving medication services after discharge from community pharmacies.

As this was a service evaluation, a purposive sampling strategy was used to recruit patients. For both surveys, patients were considered for inclusion if they were: ≥ 18 years, suffering from a LTC including: diabetes, COPD/Asthma or cardiac condition which was controlled by medication. Patients also needed to have a level of English to enable them to understand and complete the questionnaire and be capable of giving written consent to participate. An additional inclusion criterion for survey 2 was patients due to be discharged from the hospital. Patients were excluded if they were: < 18 years, unable to understand written or spoken English, with cognitive impairment, and those unwilling to participate. Sample size calculation was performed using the Raosoft sample size calculator providing a confidence level of 95% with margin of error of 5% and based on the approximate number of inpatient beds in both hospitals. St’ Georges hospital has 1300 inpatient beds and Croydon University Hospital has 565 inpatient beds. Therefore, the total sample size was 319 for each survey at 95% confidence level and 5% margin of error.

For both surveys, each patient was invited by one researcher to participate in a structured interview using a questionnaire. Patients were given a detailed information sheet about the study to read and consider before participation.

After ethical approval and prior to data collection, pilot studies were conducted to validate the two surveys involving 35 and 14 patients, respectively.

### Data analysis

All responses to both questionnaires were coded and entered into Microsoft excel spreadsheets and then analysed using Microsoft Excel as well. Simple descriptive statistics was used to summarise the demographic characteristics of the participants and the generated findings and valid percentages were reported.

### The qualitative phase: Semi-structured interviews with patients

This phase was undertaken to address the second aim of this study. A purposive sampling strategy was used to recruit participants, by approaching patients attending the respiratory HoT clinic at Croydon University Hospital. This strategy was employed to ensure the feasibility and convenience of face-to-face interviews with patients. Patients were approached in person by two researchers (NA, MAU) at the respiratory HoT clinic and provided with a detailed information sheet about the study to read and consider. Patients were included in the study if they: have been diagnosed with COPD for at least one year, and on current COPD medications. Patients unable to speak or understand English were excluded. Patients who met the inclusion criteria and accepted to participate were included. The interview schedule (See Additional file 2) was designed by the authors and included 23 open and closed ended questions that were divided to cover the following areas: demographics, disease knowledge, decision making and counselling, use of Patient information Leaflet (PIL), and awareness and experience of the MUR service.

Sample size was guided by the concept of data saturation [23, 24], which refers to the point where no new information is emerging out of the interviews and hence the collection of new information does not yield any further exploration of the issue under investigation [24]. Based on a stopping criterion of 3 consecutive interviews with no new information [23], data saturation was achieved at the 15th interview. However, all interviews were included for analysis.

The interviews were carried out in the respiratory HoT clinic between January and March 2016 by two researchers (NA, MAU). Permission was granted from the specialized respiratory team running the respiratory HoT clinic to conduct the interviews at the premises. Patients were required to sign a written consent form for conducting and recording the interviews. Each interview took an average of 15 min to conduct. All interviews were audio recorded and transcribed verbatim for subsequent analysis.

### Data analysis

Thematic analysis adopting inductive/deductive approaches was used to identify key themes. The transcripts were read and re-read several times to achieve familiarization with the data, then coded manually and independently by the two researchers who conducted the interviews and a third researcher (IH) to enhance analytical rigor. The codes were then checked, discussed and confirmed by three other researchers (RK, IH and SNG) involved in the study and experienced in conducting qualitative research. Results are presented in form for themes and associated subthemes. Participants’ quotations are presented within the emergent themes and subthemes to illustrate the findings.

### Ethical consideration

Ethical approval for the quantitative and qualitative phases was granted by the Kingston University Delegated Research Ethics Committee (Ref: 1213/045). Both Croydon University Hospital and St. George’s Hospital considered the study as service evaluation and approved it as such.

## Results

### Cross-sectional survey with patients

Two short surveys were carried out at St. George’s Hospital and Croydon University Hospital. Four hundred and fifty patients were approached for the first survey and 357 patients agreed to participate, giving a response rate of 79.3%. Whereas for survey 2, 350 patients were approached and 240 patients agreed to participate, giving a response rate of 68.5%. Over 60% of patients and over a third of patients were ≥ 60 years old in survey 1 and survey 2 respectively. Diabetes, respiratory and cardiac conditions were the most commonly specified LTCs in both surveys (Table 1).

**Table 1:** Socio-demographic data

Socio-demographic Data n (%)		
Gender	Survey 1 (*N* = 357)	Survey 2 (*N* = 240)
Female	*n* = 133 (37.3%)	*n* = 100 (41.7%)
Male	*n* = 224 (62.8%)	*n* = 140 (58.3%)
Age		
18–30	*n* = 4 (1.1%)	*n* = 39 (16.3%)
31–45	*n* = 7 (2.0%)	*n* = 17 (7.1%)
46–59	*n* = 36 (10.1%)	*n* = 98 (40.1%)
≥ 60	*n* = 221(61.9%)	*n* = 86 (35.8%)
Prefer not to say	*n* = 89 (24.9%)	*n* = 0 (0%)
Main chronic condition		
COPD	*n* = 53 (14.8%)	*n* = 36 (15%)
Asthma	*n* = 45 (12.6%)	*n* = 28 (11.7%)
Hypertension	*n* = 66 (18.5%)	*n* = 40 (16.7%)
Cardiac condition	*n* = 114 (31.9%)	*n* = 70 (29.2%)
Diabetes Mellitus Types 1 & 2	*n* = 67 (18.8%)	*n* = 38 (15.8%)
Rheumatoid arthritis and osteoarthritis	*n* = 0 (0%)	*n* = 20 (8.3%)
Other LTCs, e.g.: epilepsy, chronic renal disease, inflammatory bowel disease	*n* = 12 (3.4%)	*n* = 8 (3.3%)

### Survey 1

It was noted from screening the hospital medication charts that nearly three-quarters of patients (71.1%, *n* = 254/357) had additions or changes made to their prescribed medications for the main LTC they suffer from while in hospital. However, only two-thirds of patients (66.9%, *n* = 170/254) were aware of these changes and only 40% (*n* = 68/170) were consulted about these changes. But it was found that 62.2% (*n* = 158/254) of patients wanted to be involved in the decisions about their treatment. The reasons patients gave for not wanting to be involved included “*Doctor knows best*” (*n* = 17), “*Trust in the doctor*” (*n* = 41), or expressing the feeling that the doctor is the “*expert*” (*n* = 8), did not know much about their condition and medication to take decisions (*n* = 17) and fear of learning about their condition (*n* = 5). The remaining eight patients did not provide any reasons.

### Survey 2

When asked about medication counselling received, all 240 patients said they had received medication counselling. The specific information received during the counseling were related to the following in order of frequency; how to take the medication, purpose of the medication, important side-effects action to take in case of having important side-effects, with lifestyle changes having the lowest frequency (Table 2). A third of patients thought that counselling could be improved, with an increased amount of time being the most common suggestion (Table 2).

**Table 2:** Participants’ experience with medication counselling

Experience with medication counselling		
	Number	Percentage (%)
Topics covered during medication counselling (*N* = 240)		
Purpose of the medication	239	99.5
How to take the medication	240	100
Important side-effects	92	38.3
Action to take in case of having important side-effects	42	17.5
Lifestyle changes	34	14.1
Suggestions for improvement of medication counselling (*N* = 90)		
Provision of more time	40	44.5
Change of staff	24	26.7
Amount of information provided	17	18.8
Straight forward/easy language	10	11.1
Privacy	10	11.1
Provision of medication counselling by community pharmacists after discharge (*N* = 240)		
Yes	86	35.8
No	127	52.9
Unsure	26	10.8
Provision of discharge medications from community pharmacists (*N* = 240)		
Yes	139	57.9
No	77	32.1
Unsure	24	10
Preferred sources of information if patient encountered a problem with a medication post-discharge (*N* = 240)		
GP	80	33.34
Community pharmacist	61	25.42
Patient information leaflet	58	24.16
Internet	38	15.83
Family and friends	3	1.25

The majority of patients were not aware of community pharmacy services; NMS (93%) or MUR (83%). However, once they were informed and briefed about the NMS and MUR services by the researchers, they were interested in receiving these services (84%) and (88.8%) respectively. When patients were asked where would they go if they encountered medication difficulties post-discharge, a mixture of responses was given (Table 2) with nearly a third indicating contacting a general practitioner (GP), followed by a quarter for either contacting a community pharmacist, or reading the PIL. Other responses included searching the internet and seeking help from friends and relatives.

When patients were asked directly if they were interested in receiving medication counselling post-discharge at their local pharmacy, a majority stated they were not interested (Table 2). However, the majority of patients were interested in receiving their discharge medications from a community pharmacist rather than waiting for them at the hospital (Table 2).

### Semi-structured interviews with patients

Face-to-face semi-structured interviews were performed with 18 COPD patients; 11 females and 7 males (Table 3).

**Table 3:** Characteristics of patients included for the interviews

Patients characteristics		Number of participants (out of *n* = 18)
Age group	55–65 years	5
	Over 65 years	13
Gender	Male	7
	Female	11

Analysis of the patients’ interviews revealed three main themes:

1. Lack of patient-centered care and shared decision making

Some patients expressed their frustrations as they felt that not enough attention was provided by the HCP to the specific details of their symptoms and how they were feeling in general when they were initially diagnosed, as highlighted in the below quotes:

*“I want to be treated for me, not a number in a box.”* [Patient 14]

*“It looked like they didn’t really pay much attention to how I really felt and instead went about it their own way.”* [Patient 11]

Furthermore, some participants were overwhelmed when they were first diagnosed, and this affected their ability to retain any information regarding the disease.

*“When I was first diagnosed with COPD I was kind of overwhelmed and stressed so most I didn’t recall everything said by the doctor so emm…I don’t remember if anything was said about the disease.”* [Patient 1]

In fact, all participants lacked detailed knowledge about the aetiology of the disease, but reported their awareness of smoking being the main cause of COPD.

*“Nothing was said at the beginning but I know smoking is the cause for this disease.”* [Patient 2]

*“Me and my wife were not told much about the disease so we had to do our own research.”* [Patient 4]

Analysis of the interviews also revealed that most patients (*n* = 16/18) were not simply involved in any decision making regarding the prescription of their respiratory medications.

*“No one asked for my opinion on anything [coughing]”* [Patient 2]

*“There was not really any chance for me to have any say when it came to my treatment”* [Patient 14]

Furthermore, the analysis highlighted that some patients (*n* = 6/18) did not want to be involved in any decisions regarding change to their medications as they completely rely on their doctor’s advice. Others (*n* = 8/18) felt that whatever decisions their prescriber makes, they trust them and so didn’t feel the need to try and be involved as well.

“*I don’t think I needed to be involved anyway as I already trusted what they were doing*.” [Patient 17]

*“Was not involved at all. To be honest, I only want the doctor suggestions…don’t trust myself.”* [Patient 3]

*“No involvement at all…. I prefer to rely on what the doctor decides for me.”* [Patient 7]

2- Minimal counselling provided by HCPs

Most of the patients (*n* = 14) reported that counselling and advice covered the basics such as when to take or how to take their inhalers. They felt that it was brief and that more could have been done or provided to make them fully confident about using their medicines.

*“Counselling only involved basic use of the inhalers, lasted for a few seconds.”* [Patient 6]

The rest (*n* = 4) indicated receiving no counselling when their medications were first provided.

*“I was told nothing after I received my medicines”.* [Patient 12]

Patients who received counselling reported having such counselling either from the GP or community pharmacist; leaflets were the main source of information that was given to them.

*“I was simply advised to read up in the information leaflet in order to know about my medicines”* [Patient 14]

Interestingly, most patients reported using the PIL provided with the medication before taking it. Patients reported reading the PIL to enquire more about several issues related to their medications such as: how to take/use the medicine, side effects, alcohol intake while taking their medication and check if the medicine affects driving.

*“I always make sure to have a proper look at the leaflet for anything I need to be aware of.”* [Patient 18]

3- Limited awareness about the MUR service

Only 5 patients were aware about the MUR service that is provided by the community pharmacist, and only 3 of them undertook the review with the community pharmacist. Therefore, the majority of the patients were not aware of the service and required explanation about the service. Even after providing an explanation to them, they said that they were not aware of the service and its existence at their local pharmacy.

*“No, I’ve never heard about it.”* [Patient 1]

*“I never knew this service was available, nobody told me about it.”* [Patient 12]

## Discussion

The current study sheds light on several issues related to patients’ perceptions regarding counselling about medications and SDM and their awareness of community pharmacy services that could enhance understanding of their medications and conditions. The generated results clearly identified that there is a lack of detailed medication counselling and patients’ involvement in SDM, which echo previous findings in the literature [5, 25]. Underutilisation of community pharmacy services was also highlighted in this study by the lack of awareness among target patients about services related to medication optimisation and counselling which can be promptly and easily accessible at primary care level. A new finding revealed in this study was patients’ preference for receiving their discharge medications from community pharmacies. This could provide a solution for the delay in discharge related to the long wait for discharge medications highlighted in the literature [25].

This study highlighted problematic issues related to counselling in both primary care and during transition of care. Despite the fact that all patients have received medication counselling upon discharge, yet all consultations only covered basic topics mainly the purpose of the medication and how to take it, whereas other important topics related to side effects and life style changes were only covered in less than 40% of these consultations. More than 30% of patients who participated in the second survey thought that medication counselling could be improved prior to hospital discharge. Interestingly, in previous research, patient counselling was perceived by HCPs to be limited at hospital discharge in England [5]. Counselling was also perceived by the interviewees to be basic, brief and minimal. Patients expressed their need for more detailed counselling during the interviews. Not surprisingly, this study found that a quarter of the surveyed patients would refer to the PIL if encountered problems with a medication post-discharge. This was echoed by the interview results where interviewees reported the use of the PIL to seek additional medication information, which can be related to the minimal counselling reported by these patients. In previous research, the main reason cited by patients for not reading PILs was the provision of previous effective counselling by HCPs [26].

The need for better education about COPD as a disease among patients has been previously raised in the literature [8, 27, 28] to improve patients’ quality of life [8]. Despite that patient education has been acknowledged as pivotal to the care of COPD patients and a cornerstone to the management of the disease [8, 27, 29], yet the current study still highlights basic knowledge about the disease. This indicates that nothing has changed with respect to patient education, despite the latter being reported as poor among COPD patients in the literature for more than 10 years [30].

The results of the first survey showed that although 70% of patients had changes made to their medications, only a third was consulted about the changes. The same was echoed in our interviews, which denotes a paternalistic model of care rather than SDM. In addition, the interviewees highlighted lack of personalised care by HCPs. This finding is of concern, given the increased advocacy in the National Health Service (NHS) in England towards the implementation of patient-centred care which involves SDM [31]. Previous studies in England highlighted lack of patient involvement throughout the discharge process [5, 25] and primary care consultations [32]. For over 25 years, NHS directives have recommended increasing patients’ involvement in their care decisions [33]. This was encouraged from the 1990’s [33–35] and in 2012 the phrase “No decision about me, without me” [36] was devised to advertise the NHS commitment to SDM, so that this becomes the norm practice across the NHS [25, 36]. Yet, the current results show that SDM is still not widely implemented during consultations with patients.

Additionally, more than two-thirds of patients at the two hospitals wanted to be involved in decisions about their medications. Patients who did not want to be involved in their treatment expressed trust in the doctors’ decision; this confidence in doctors was shown again by patients’ HCP preference for receiving medical information. However, this was different in our interviews, as most of the interviewees were not willing to be involved in decisions about their care. What was clear from the interviews is that some patients did not want to be involved in their care, whereas others did not perceive such involvement to be important. However, patients’ preferences for participation in decision making can vary substantially, hence patients can either adopt a passive role or an active/collaborative role in making decisions about their health [37, 38]. Research conducted in the USA, showed that although patients described participation in decision making, yet many deferred the final decision to their doctors [39]. Another study in England showed that one third of the patients preferred the doctors to make decisions for them [40]. However, provision of patient-centred care involving SDM necessitates different HCP skills, maintaining patient trust, and the provision of detailed counselling including evidence-based information about options, outcomes and uncertainties, together with decision-support counselling [41, 42]. Hence, the provision of brief and minimal counselling reported in this study provides a potential explanation to the reported lack of personalised care and SDM experienced by the interviewees. Another possible explanation would be age; most of the interviewees in the current study were over 65; which was reported in the literature to affect patients’ preferences for SDM [15, 43], with older patients preferring to be less active in medical decision making during consultations in primary care [15]. Despite age being also cited as a reason for patients being passive in decisions about care during the hospital discharge process [44], yet most patients at the two hospitals, despite being elderly, wanted to be involved in decisions. Interestingly, this highlights a difference for SDM preference which can be related to the healthcare setting in this study. In the hospital setting and specifically during hospital discharge which is a complex and lengthy process [25], SDM was more preferred than in the respiratory HoT clinic setting which was developed in Croydon to provide a smooth and rapid service in the community to high-risk patients. In fact, patient involvement is a complex and multifactorial issue which can be affected by patient, doctor and contextual factors such as clinical settings [45]. This in return re-emphasises the need for HCPs to adopt interventions to promote patient participation including elderly through creating a trusting environment during the consultation that allow patients to feel comfortable speaking up and asking questions about their care [15], given the increased prevalence of elderly people with LTCs [14]. In one study, the doctor’s communicative style was found to be one of the strongest predictors of patient involvement in care [45].

Despite the MUR service being introduced in England in 2005, the current findings still highlight a considerable lack of awareness of the service among participants in both phases of the study. This finding is of also of concern due to several reasons. First, the MUR service has been introduced for more than 10 years in the community pharmacy sector in England. Second, community pharmacies in England are expected to perform at least 70% of MURs with patients falling within the agreed national target groups since April 2015 [46]. Post-discharge and respiratory patients having COPD are among the national target groups for this service [46]. Research evidence from several countries demonstrated low awareness of medicine related cognitive services offered by community pharmacists among the public [11]. A survey study in 2012 among the public and community pharmacists in England reported low awareness of the MUR service among the public, even in regular medicines users [11]. The latter study also highlighted how community pharmacists overestimated patients’ awareness of the MUR service, in contrast to the actual awareness reported in the study which was indeed low [11]. The current results also show a remarkable low level of awareness among patients who are the target groups for this service. This indicates that nothing has changed to promote patients’ awareness about services that are easily accessible and essential to patients with LTCs such as the MUR service in the community pharmacies in England. This also places a great emphasis on the importance and need among all HCPs, whether in primary or secondary care to promote awareness especially among target patients about the MUR service in England. It also aligns with the call raised by the Royal Pharmaceutical Society in the UK for greater public awareness of pharmacy services [47]. In the current study, patients at both hospitals wanted to participate in MUR and NMS services once informed about them. However, a study in England highlighted that few hospitals refer patients after discharge to community pharmacies for MUR or NMS as currently recommended [5], the research also highlighted the lack of communications between HCPs in secondary care with HCPs in primary care especially community pharmacists. In addition, existing evidence suggests that patients still consider GPs as their main source of information about their medications after discharge, which might be due lack of awareness about the MUR and NMS services at the community [25], hence patients can be more willing to participate in these services if they are signposted by their GPs [13]. Patients at the two hospitals also wanted to receive their discharge medications from community pharmacists, possibly due to the long waiting times in hospitals. In a previous research in the UK, waiting for medicines was the most commonly perceived reason for delays in hospital discharges [25]. A transfer of the provision of discharge as well as of out-patient medications from hospital to local community pharmacies, as proposed in the Carter report [48] would enable patients to receive their medications and additionally NMS and MUR at a more convenient location and time. This also coincides with the calls to provide a new model of care for patient discharge which involves support with medicines after discharge by community pharmacists [25].

The current study has several limitations including the small sample size and the use of purposive sampling within an urban community in London borough of Croydon for the qualitative phase. Interviews were sought among patients with one LTC which is COPD; hence the results may not be reflective of the views of patients with other LTCs. Patients with COPD were chosen for this phase for three main reasons. First, the convenience of recruitment to the researchers within the respiratory HoT clinic at Croydon University Hospital. Second, COPD patients are a target group for MUR and NMS services offered in community pharmacies. Third, the high economic burden associated with COPD treatment with respect to exacerbations and hospital admissions.

In addition, as patients were in acute setting, the data collection tool for the quantitative phase had to be concise, hence the topics evaluated had to be split into two surveys. Even though minor amendments were required with the surveys, yet the pilot surveys were not included in the final analysis to avoid any type of bias. The sample size required for survey 2 was not achieved despite approaching 350 patients; this could be due to the fact that the survey was done among patients ready to be discharged from the hospital so this may have impacted their willingness to complete the survey in order to avoid delays in leaving the hospital. Another limitation is that population age in the two surveys was different; as older patients were more prevalent in the first survey, whereas younger patients were over presented in the second survey.

## Conclusion

A significant number of patients are still not involved in discussions about changes to their medication/treatment despite many NHS recommendations to move from a paternalistic approach to a shared decision model. Despite the huge cost associated with the poor management of COPD and patients post-discharge, and the fact that SDM could support patients’ management in relation to medicine optimisation, exacerbations and hospital admissions/re-admissions, the principle is not widely implemented. There was also a remarkable low level of awareness of the MUR service among post-discharge and respiratory patients who are target groups for this service. The current results emphasise the need among HCPs in primary and secondary care to promote awareness among patients about services targeting medicine optimisation, regardless when these services were introduced, in order to enhance care experienced by patients with LTCs. It also reinforces the need among HCPs in primary and secondary care to promote patient-centred approach during consultations to enhance SDM. This study is limited to two hospital settings in Greater London, a similar study on a national scale is recommended to further investigate patients’ preference for receiving discharge medications from community pharmacies among larger population and investigate the feasibility of such a new model of post-discharge service provision to enhance the quality of care in the future. Nevertheless, using community pharmacy services for medication supply and counselling post discharge could provide a solution to the pressure experienced by acute NHS hospitals [49] and hence increase their operational productivity and performance in the future, and comes in line with the government’s health policy “Better care Fund” to rely more on community-based services in the future [49, 50].

## Additional files

Additional file 1: The quantitative surveys. (DOCX 105 kb)

Additional file 2: The interview schedule. (DOCX 22 kb)

## Abbreviations

COPD: Chronic Obstructive pulmonary disease
GP: General practitioner
HCPs: Healthcare professionals
LTCs: Long term conditions
MUR: Medicine Use Review
NHS: National Health Service
NICE: National Institute for Health and Clinical Excellence
NMS: New Medicine Service
PIL: Patient information Leaflet
SDM: Shared decision making
