# Supplementary material for: Shared decision making and experiences of patients with long-term conditions: has anything changed?
Source: BMC Health Serv Res. 2018 Oct 10;18:763. doi: 10.1186/s12913-018-3575-y (PMC6180612; doi:10.1186/s12913-018-3575-y)
Supplement: Supplementary file 1 — The quantitative surveys. (DOCX 105 kb) [file 12913_2018_3575_MOESM1_ESM.docx]

# The quantitative surveys (1) and (2)

## Questionnaire for Survey (1):

**Section A: Opinion on Shared Decision Making and involvement in decisions about changes to treatment/ medications**

**Section B: Opinion about Services**

| 1. Are you aware of the concept of shared decision making? | YES NO |
| --- | --- |
| 2. Were you aware of any changes that have been made to your medications upon admission to the hospital? | YES NO |
| 3. Were you consulted regarding changes made to your medications? | YES NO |
| 4. Were you involved in the decision to change your medications? | YES NO |
| 5. Were you consulted regarding your discharge time? | YES NO |
| 6. Were you consulted on how your care/treatment plans would be after you are discharged from the hospital? | YES NO |
| 7. As a patient do you want to be involved in the decision to change your medications? | YES NO |
| 8. If you would NOT like to be involved in making decisions about your treatment, would you please say why not? |  |

**Section B: Demographic**

| 1 .Gender | Female  Male |
| --- | --- |
|  |  |
| 2. Age |  |
|  | ≤ 30 |
|  | 31-45 |
|  | 46-59 |
|  | ≥60 |

## Questionnaire for Survey 2

St George’s hospital and Croydon University Hospital are carrying out a study to determine whether patients are satisfied with the medication counselling that they receive when discharged from our hospital. Your experiences and opinions are of great value to us. We aim to use the results of this study to improve the quality of the counselling services provided to patients. We would be very grateful if you could take a few minutes of your time to complete this questionnaire.

| Section 1: Your Medication Counselling Experience | | | | |
| --- | --- | --- | --- | --- |
|  |  | **Please tick all boxes that apply** | |  |
| 1 | **Did you receive any medication counselling?** | **Yes**  **No**  **Unsure** | |  |
| 2 | **What specific information did you receive during your counselling?** | **Purpose of medicine**  **How to take the medicine**  **Important side-effects**  **Action to take following an important side-effect**  **Lifestyle changes**  **Alternative medications and therapy**  **Other** (please specify in adjacent column) | |  |
| 3 | **How could the medication counselling be improved?** | **More privacy** | |  |
|  |  | **Change of staff**  (Please specify who you would prefer to be counselled by) | |  |
|  |  | **Different form of information provided** | |  |
|  |  | **More straightforward language** | |  |
|  |  | **Amount of information**  (please specify if more or less) | |  |
|  |  | **Amount of time taken**  (please specify if more or less) | |  |
|  |  | **Other** (please specify in adjacent column) | |  |
|  |  | **None of the above** | |  |
| Section 2: Other sources of information | | | | |
| 7 | **If you encounter problems with your medications after discharge, where would you get medicines information from?** | **The Internet**  **GP**  **Community pharmacist**  **Patient information leaflet**  **Relatives and friends**  **Other** (Please specify) |  | |
| 8 | **Following discharge, would you be interested in receiving your medication counselling from your local community pharmacist?** | **Yes**  **No**  **Unsure** |  | |
| 9 | **Following discharge, would you be interested in receiving all of your medications from your local community pharmacist?** | **Yes**  **No**  **Unsure** |  | |
| 10 | **If a community pharmacist offered home delivery of your discharge medications, would you be interested in using this service?** | **Yes**  **No**  **Unsure** |  | |
| 11 | **Are you aware of a patient counselling service offered by community pharmacists to discharged patients called the “targeted MUR”?(Explain)** | **Yes**  **No**  **Unsure** |  | |
| 12 | **Would you be interested in receiving this service?** | **Yes**  **No**  **Unsure** |  | |
| 13 | **Are you aware of a patient counselling service offered by community pharmacist for patients who have been newly prescribed certain medications called NMS?(Explain)** | **Yes**  **No**  **Unsure** |  | |
| 14 | **Would you be interested in receiving this service?** | **Yes**  **No**  **Unsure** |  | |
| Section 3: Demographics | | | | |
| \| **15.Gender** \| **Female**  **Male** \| \| --- \| --- \| \|  \| \| **16. Age** \|  \| \|  \| **≤ 30** \| \|  \| **31-45** \| \|  \| **46-59** \| \|  \| **≥60** \| | | | | |
