# Supplementary material for: Shared decision making and experiences of patients with long-term conditions: has anything changed?
Source: BMC Health Serv Res. 2018 Oct 10;18:763. doi: 10.1186/s12913-018-3575-y (PMC6180612; doi:10.1186/s12913-018-3575-y)
Supplement: Supplementary file 2 — The interview schedule. (DOCX 22 kb) [file 12913_2018_3575_MOESM2_ESM.docx]

# The interview Schedule

**Disease Knowledge**

1. When were you diagnosed?
2. What were you told about the disease?
3. Have you made any life style changes? (Prompt: smoking cessation)
4. Do you feel the people around you know enough about your disease?

**Decision making and counselling**

1. If you could go backward to the time your medication was first prescribed or a change has been made, can you tell me whether you were involved in the decision regarding your respiratory medication being prescribed?
2. What information was provided to you?
3. When the medication was prescribed or dispensed to you, did you receive any counselling?
4. What did that counselling involve?
5. What source of information were you given? (Prompt: handouts, leaflets, posters)
6. How was it provided to you? And by whom?
7. At what stage was the information provided to you?
8. What other information, if any, you would have liked to receive and how?

**Patient Information Leaflet (PIL)**

1. Do you use Patient Information Leaflet? Yes No

If response is “Yes”, continue to Q14 and Q15

If response is “No”, go to Q16

1. How useful did you find the information given in PIL?
2. What information did you get from the PIL?

**Medicine Use Review (MUR)**

**Community pharmacists are offering MUR service that aims to help patients get the most out of their prescribed medicines for long term conditions such as COPD.**

1. Are you aware of the MUR service? Yes No

If response is “Yes”, continue to Q17

If response is “No”, go to Q22

1. Have you ever had a review with the Community pharmacist? Yes No

If response is “Yes”, continue to Q18-Q23

If response is “No”, go to Q22

1. What source of information was provided during that review?
2. How did this service improve your knowledge about your medication and the use of your medication?
3. What information was given to you during the MUR review?
4. How do you feel about the information given during your MUR regarding your medication?

**Demographics**

1. What is your age?

Under 30 35-45 45-55 55-65 +65

1. What is your gender? Female Male

Do You have any questions that you would like to ask me?
